# Spread of SARS-CoV-2 at school through the pandemic waves: a population-based cohort study in Italy: Marco Fonzo

**DOI:** 10.1093/eurpub/ckac129.281

**Published:** 2022-10-25

**Authors:** M Fonzo, I Pistellato, A Calzavara, P Sorrentino, V Selle, LG Sbrogiò, C Bertoncello

**Affiliations:** 1 Hygiene and Public Health Unit, DCTVSP, University, Padua, Italy; 2 Department of Prevention, Local Health Authority, Venice, Italy

## Abstract

**Background:**

To limit SARS-CoV-2 transmission, proactive closure of schools is often believed by policy-makers and public an effective strategy. While evidence on the role of students in the spread is ongoing, effects of closure on children's well-being are well known. The number of secondary cases per class has been considered one of main driving criteria to mandate for distance learning. We aimed to calculate the rate of secondary infections per classroom and to identify factors associated with the development of school clusters.

**Methods:**

We conducted a population-based cohort study between October 2020 and November 2021 in the province of Venice, Italy, a catchment area of 600,000 inhabitants. Primary, middle and high-schools were included.

**Results:**

We identified 1,623 primary cases of SARS-CoV-2 infection in students. Of these, 72.5% did not lead to any secondary case in the school setting, 15.6% to 1, and 11.9% to 2+ contagions. The so-called second wave (Oct-Dec 2020) was associated with a lower occurrence of 2+ contagions (AOR=0.37; 95%CI: 0.24-0.56) than the fourth (Sep-Nov 2021). Both primary (AOR=1.74; 95%CI: 1.16-2.63) and middle schools (AOR=1.76 95%CI: 1,14-2,72) showed higher odds than high schools for cluster generation of 2+ cases. The involvement of 2+ secondary cases was lesser associated with the index case being a student rather than school staff (AOR=0.42; 95%CI: 0.29-0.60). The number of 2+ cases clusters per week followed a time trend in line with the general population incidence.

**Conclusions:**

The school environment does not facilitate viral spread, but rather reflects transmission in the community. Appropriate measures (use of airway protection devices, interpersonal distancing, frequent hand and respiratory hygiene) and timely case tracking make school a safe place. Given the documented negative effects of school closures on children's learning and well-being, maintaining school attendance is as essential as it is desirable.

**Key messages:**

• A SARS-CoV-2 positive student at school does not generate secondary infections in 3 out of 4 cases. The risk of cluster generation is lower when the index case is a student rather than school staff.

• The school environment does not facilitate viral spread, but rather reflects transmission in the community. School attendance is essential considering the effects on children’s learning and wellbeing.

